# Commentary on: Practice Management Knowledge Amongst Plastic Surgery Residents in Canada: A National Survey

**DOI:** 10.1093/asjof/ojaa040

**Published:** 2020-08-12

**Authors:** Jamil Ahmad, Frank Lista

It is with great pleasure that we discuss “Practice Management Knowledge Amongst Plastic Surgery Residents in Canada: A National Survey” by Hong et al.^1^ In this article, Hong et al attempted to assess the business and practice management teaching experience amongst plastic surgery residency programs in Canada. They designed an online survey with 8 questions to examine 4 main areas: (1) demographic information, (2) business education and experience before residency training, (3) business and practice management education during residency training, and (4) self-reported knowledge about core business principles. Participants were asked to rate their confidence and knowledge in 8 areas of business and practice management using a 10-point Likert scale with 1 representing lack of knowledge and confidence and 10 representing complete knowledge and confidence. These topic areas were identified by Zarrabi et al^2^ in their business of healthcare curriculum as core components of practice management relevant to surgical training. The 8 topic areas include branding and marketing communication, leading healthcare organizations, effective teams, negotiation, accounting/financial reporting/billing/reimbursement, business communication, strategic human capital, and business law/legal and governance.^2^ The survey was sent to all English-speaking Canadian plastic surgery residency programs. The survey response rate was 51.6% with 65 of 126 residents responding. Not surprisingly, only 7.8% of participants had previous business and practice management training; 23.1% reported receiving training in business and practice management during their residency. Participants reported a low level of knowledge and confidence in business and practice management and a high desire for future training in business and practice management particularly in billing and coding (91.2%) and business operations (91.2%).

There are several obstacles to improving business and practice management training for plastic surgery residents:

1. Time—Both depth of knowledge and breadth of practice in plastic surgery continue to increase but work hour restrictions in postgraduate medical education make it challenging to provide adequate and comprehensive clinical exposure and teaching in all areas including business and practice management required to competently practice.
2. Expertise—Faculty and others involved in teaching may not have sufficient expertise in all areas of business and practice management to teach this effectively to postgraduate trainees.
3. Practical Exposure—The varying practice models included in the clinical experience in a postgraduate training program at an academic institution and its affiliated medical centers may not be representative of the practice model that a postgraduate trainee may have once in practice.

Business is playing an increasingly more important role in the practice of medicine.^3^ Regardless of their practice model, physicians need fundamental knowledge and skills in 3 key business disciplines: leadership, teamwork, and data analytics.^4^ An active effort to incorporate education in these critical areas in postgraduate plastic surgery training programs is required. Starting July 2020 with the Competence by Design medical education model, Canadian plastic surgery residents will progress through several stages in their training, including Transition to Discipline, Foundations of Discipline, Core Discipline, and Transition to Practice.^5^ Within each of these stages, there are required, recommended, and optional training experiences. These training experiences involve clinical experiences and other training experiences, such as formal instruction, surgical skills, certifications, and examinations. Despite this framework including focused training experiences such as formal instruction, much of the knowledge and skills acquired in residency are during day-to-day clinical activities—learning on the job. Observational learning and modeling play a significant role in knowledge and skill acquisition in plastic surgery so that exposure and involvement of plastic surgery residents to the business and practice management aspects of practice are necessary.

Creating opportunities for the comprehensive management of the patient’s journey through their care pathway exposes the resident to nonclinical aspects that the resident may not commonly experience. In our plastic surgery residency training program at the University of Toronto, one of the best opportunities for this has been the resident aesthetic clinic where residents are not only responsible for the clinical aspects of care but also expected to coordinate and schedule patient care with office staff, order some medical supplies from suppliers, learn about relationships with industry, practice billing, and understand the costs involved in managing a plastic surgery practice. Our residents have found this to be an unique and invaluable experience.

In this study, “billing and coding” was the most highly prioritized of the 8 core business principles by Canadian plastic surgery residents. This concept can be easily incorporated into day-to-day clinical activities with residents performing shadow billing and reviewing these with faculty. Residents should also be taught the necessary elements required to be documented in the medical record to justify the billing of services rendered.

One of the more challenging areas for postgraduate training programs to provide exposure is in human resources and employment standards. Beyond formal instruction, it is difficult to provide focused practical experience. However, focusing on the development of leadership, teamwork, and communication skills, combined with understanding legal aspects of managing employees, will give the plastic surgery resident a foundation for continuing to develop these personnel management skills in their future practices.

Beyond formal postgraduate medical training, there continue to be opportunities to further enhance business and practice management knowledge. Professional societies such as the Aesthetic Society offer a significant amount of educational content covering all the aspects of business and practice management. At the annual Aesthetic Meeting, specific educational programming on business and practice management is provided for both physicians and their staff. To help plastic surgeons gain knowledge and experience earlier in their careers, the Aesthetic Society offers the annual Residents’ Symposium, which focuses on career development and business and practice management training. For Aesthetic Society members, the Aesthetic Society Leadership Development Program focuses on developing leadership and teamwork skills, which are critical to be successful in practice and involvement with various organizations—unfortunately, experiences such as this are not common in formal postgraduate medical training. Additionally, companies exist within the plastic surgery space to provide services and education to plastic surgeons and their practices to help improve their business and practice management processes.

In summary, this article provides us insight into the perspectives of our Canadian plastic surgery residents about their educational experience with business and practice management during their postgraduate training. It confirms what many of us already know—although business and practice management skills are critical to the success of a plastic surgeon’s practice, our plastic surgery residents are not receiving enough education in this area to feel confident to start their practices and manage their businesses. Curriculum redesign and adoption of a competency-based medical education framework offer an opportunity to incorporate essential business and practice management educational experiences in Canadian plastic surgery residency training programs. However, this will require a measured and thoughtful approach to provide these educational experiences in the most effective and efficient manner given the aforementioned constraints.
